# Buffalo General Hospital

**Published:** 1876-03

**Authors:** Charles C. F. Gay

**Affiliations:** Attending Surgeon


ART. IV.—*Buffalo General Hospital. Notes of Surgical cases.* By CHARLES C. F. GAY, M. D., Attending Surgeon.

FIBROUS POLYPUS OF THE NOSE. AVULSION BY A NEW METHOD. —Mrs. ——, aged 35 years, entered Hospital April 30, 1875. She had a large fibrous polypus of the right naris from which she had frequent hemorrhages ; bleeding is provoked by the most delicate touch with the silver probe. Its attachment was far back toward the posterior nares. I passed the fore-finger of my right hand into tne anterior naris, crowded the polypus up and back through the posterior nasis, and with the finger nail detached the quite firm connections of the tumour from the turbinated bone ; when it dropped back—a large mass—into the mouth. The finger was retained in the naris for a moment after the polypus had escaped.

REMARKS.—Avulsion by this method was quickly accomplished and hemorrhage was avoided. A few weeks before this case presented itself I had removed a nasal polypus, or a large portion of it, by torsion with the forceps. The hemorrhage following the operation was profuse, and not having means within reach to arrest it, I immediately thrust my fore-finger, its entire length into the naris, holding it there a few minutes, when, on its withdrawal, all bleeding had ceased. It was this experience that led me to adopt the method for avulsion in the case now at hand.

The writer does not claim this method as the best by any means, for the removal of nasal polypi, but it answered the purpose in this case admirably. The mode is applicable, I think, to all cases of gelatinous and cystic nasal tumours, and in cases of fibrous tumors with even strong and ancient attachments; it ought, in some cases to be tried before resorting to other modes which are so often vexatious and unsatisfactory. The objection that the detached tumour might perchance lodge where it would produce death by suffocation, is really no objection at all, provided the tumour be large and the Surgeon on his guard against any such danger.

Curvature and Hypertrophy of the Nasal Septum.—This deformity existed in a female, aged 22 years. The right naris was entirely occluded and besides the constant annoyance which this caused, there was also existing an unsightly malposition of the nâsal septum; a bulging out upon the right side with correspondent depression on the left side the nose. So unsightly and troublesome was the condition of this patient's nose, that no urging was required to induce her to submit to the ordeal of any surgical operation that promised improvement.

Accordingly, chloroform was given and an incision made close upon the border of the septum about three-quarters of an inch in length, thus laying open the cavity of the naris. The cartilaginous portion of the septum was now pared down fully one-quarter of an inch, care being taken not to perforate the septum. The wound was dressed and united by primary union; the deformity was much relieved, air passed up through the nostril, but all the advantage that could be desired, from the operation, was not obtained on account of the hypertrophic condition extending so far back as not to be easily reached with the knife.

Morbus Coxæ.—Resection of Head and Neck of the Femur. Result.—A boy, aged seven years, entered the Hospital April 11th, 1875, with morbus Coxæ, of two years duration. The cause of the hip-joint disease was supposed to be of traumatic origin; from a fall down stairs, since the boy complained of pain which he referred to the hip aftêr the fall. There are three sinuses reaching down to the left hip joint. Suppuration has been, and is now copious. The boy is emaciated from the purulent discharge; has suffered much pain, especially when moved, and has been confined to his bed for a long time. He has been taking Quinine gr. j. three times daily with cod liver oil.

On May 25th the boy, from use of the tonic treatment, had so much improved, as to warant the performance of an operation for resection. This was done in the presence of most of the members of the Medical and Surgical Staff of the Hospital in the following manner : I made a longitudinal incision in the axis of the head and neck of the bone about three inches in length, beginning from just above the trochanter major, cutting at once down upon the bone, separating the soft parts from it, then passed around the bone, between the greater and lesser trochanter, the chain saw and divided the bone. The section of bone thus separated from the shaft was seized with forceps and removed from the acetabulum- A small portion of the head of the bone remained in its socket and was removed with the scoop.

It was then ascertained that the acetabulum was more or less im. implicated in the disease. It was somewhat carious. Its snrface was eroded and rendered the prognosis unfavorable.

The three inches of femur removed was eroded and carious.

The wound was left open and water dressings applied. Chloroform was used and its effect maintained by ether. To our great surprise this boy made a good recovery.

From the time of the operation until convalescence he enjoyed immunity from pain, became fleshy, and on Oetober 1st, 1875, had obtained use of the limb and aided by crutches he could walk. He has a good hip joint, can rest his weight upon the limb, can move it at will in any direction. The shortening is one and a half inches.

A few days after the operation, and after subsidence of inflammatory action, extension was made and the boy put into wire breeches with the leg brought into the straight position.

Hallux Valgus.—Resection of half an inch of the head of the first mettarsal bone for abductiou of the great toe is a recent operation; I have performed it twice at the Hospital during the past year.

I made use of Esmarch's bandage, made a single longitudinal incision over and of equal distance above and below the metatarso-phalangial articulation, one inch and a half in length, and removed the head of the bone by the chain saw. When the bone is removed the phalangial bone drops into line with the axis of the foot and the case needs no after treatment save the warm water fomentations.

The relief of the deformity is absolutely perfect. With the use of Esmarch's bandage not a drop of blood need be lost. Pressure upon the wound with a sponge or compress for a few minutes will prevent loss of blood from the returning current when the bandage is removed.

It will not be mal-apropo to remark in this connection that Esmarch's bandage is of the greatest service and indeed is indispensable in the search for pieces of broken needles that are embedded in the muscles of the hand or fore-arm. I have removed pieces of broken needles from the hand and wrist with the greatest facility with the help of Esmarch's bandage when it was found to be impossible without it to find the foreign body; when, indeed the embedded needles had eluded the search for more than a year of those who had made the attempt to find it; hence Esmarch's bandage lends to some of the minor operations of surgery as much interest as attaches sometimes to the more formidable operations.

